# Action 3:30R: process evaluation of a cluster randomised feasibility study of a revised teaching assistant-led extracurricular physical activity intervention for 8 to 10 year olds

**DOI:** 10.1186/s12889-019-7347-3

**Published:** 2019-08-14

**Authors:** Byron Tibbitts, Alice Porter, Simon J. Sebire, Emma L. Bird, Emily Sanderson, Chris Metcalfe, Jane E. Powell, Russell Jago

**Affiliations:** 1 Centre for Exercise, Nutrition & Health Sciences, School for Policy Studies, University of Bristol, 8 Priory Road, Bristol, BS8 1TZ UK; 2 Bristol Randomised Trials Collaboration, Bristol Trials Centre, University of Bristol, Bristol, BS8 2PS UK; 3 Centre for Public Health and Wellbeing, University of the West of England, Bristol, BS16 1QY UK; 4 The National Institute for Health Research Collaboration for Leadership, Applied Health Research and Care West (NIHR CLAHRC West), University Hospitals Bristol NHS Foundation Trust, Bristol, BS1 2NT UK; 5 Present address: Population Health Sciences, Bristol Medical School, University of Bristol, Oakfield House, Oakfield Grove, Bristol, BS8 2BN UK

**Keywords:** Physical activity, Children, Teaching assistants, Feasibility, Intervention, After-school, Process evaluation, RE-AIM

## Abstract

**Background:**

Numerous interventions to increase children’s physical activity levels are published, yet, few studies report indicators of external validity. Process evaluations are critical for assessing intervention implementation, sustainability and effectiveness. A mixed-methods process evaluation, using the RE-AIM framework, was conducted to evaluate the internal and external validity of Action 3:30R, a revised teaching assistant-led after-school intervention which aimed to increase physical activity in children aged 8–10 years and was underpinned by Self-determination Theory (SDT).

**Methods:**

Data were collected and reported in line with the five components of RE-AIM (*Reach, Effectiveness, Adoption, Implementation* and *Maintenance*). Quantitative measures included logbooks, registers and self-reported teaching-efficacy, autonomy support, child enjoyment and perceived exertion questionnaires. Questionnaire data were collected at three points throughout the 15-week intervention. Observations by trained researchers were also conducted to assess fidelity to the intervention manual and its underpinning theory. Post-intervention focus groups with pupils and interviews with teaching assistants (TAs), school staff and external stakeholders explored the implementation and potential sustainability of Action 3:30R from stakeholders’ perspectives.

**Results:**

Action 3:30R appealed to a broad range of pupils, including girls and less-active pupils. The Action 3:30R TA training was implemented as intended and was perceived as valuable professional development. Releasing staff for training was a barrier in two of the six intervention schools, which were unable to deliver the intervention as a result. Pupils enjoyed the intervention, and the Action 3:30R core principles underpinned by SDT were implemented with high fidelity, as was the intervention itself. Scheduling conflicts with other clubs and lack of parental support were perceived as the main barriers to recruitment and attendance. Lack of space and season were cited as the main barriers affecting the quality of delivery. The study shows evidence of maintenance, as one intervention school decided to continue Action 3:30R beyond the study. Funding and continued TA training were suggested as factors which may affect the maintenance of Action 3:30R.

**Conclusions:**

Action 3:30R is an enjoyable, autonomy-supportive after-school programme, which engages a range of pupils and offers TAs valuable training. RE-AIM provided helpful structure and is recommended for intervention evaluations.

**Trial registration:**

ISRCTN34001941. Prospectively registered 01/12/2016.

**Electronic supplementary material:**

The online version of this article (10.1186/s12889-019-7347-3) contains supplementary material, which is available to authorized users.

## Background

Physical activity is associated with improved physical and mental health in young people [1, 2] but 49% of children do not meet the recommended 60 min of moderate to vigorous physical activity (MVPA) per day [3]. Children are less likely to meet the recommendation if they are inactive after the school day [4]. Schools are important settings in which to embed programmes which promote and provide opportunities for physical activity [5]. Teaching assistants (TAs) play a significant role in the school system by supporting teachers in class management and teaching. Training TAs to deliver after-school physical activity programmes could be an effective way for primary schools to utilise their existing staff, use their Physical Education (PE) and Sport Premium (ring-fenced funding currently offered by the UK government to primary schools to improve the quality of PE and sport offered to pupils) and enhance or offer more physical activity provision [6]. The Action 3:30R study aimed to assess the feasibility, cost-effectiveness and potential sustainability of a revised after-school physical activity intervention delivered by TAs.

Numerous physical activity interventions in children have shown promise at pilot/feasibility stage but failed to achieve an impact in a definitive trial [7, 8], or if an impact was achieved in a trial it was not sustained during implementation [9]. Interventions in educational settings are often very complex, and potentially affected by contextual factors beyond the focus of the outcome evaluation. Process evaluations enable exploration of these factors, and can greatly enhance understanding of any findings focussed on effects – specifically the mechanisms of impact [10]. They are important for understanding whether interventions have been delivered as intended, factors which may have affected delivery and the potential sustainability [11]. Conducting a process evaluation also helps with generalisability and translating research into practice [12].

The RE-AIM evaluation framework was developed to understand and measure the potential population health impact of an intervention [13]. RE-AIM comprises five components: Reach, Effectiveness, Adoption, Implementation, Maintenance. Exploring *reach* and *adoption* can speak directly to the external validity of a trial as they account for the representativeness and uptake across a target population at both an individual and a cluster-unit level. *Effectiveness* investigates mechanisms of impact, both in relation to the main outcome of a trial and other effects on participants not investigated in the main trial. *Implementation* is concerned with fidelity of delivery, and therefore internal validity, and exploring factors affecting *maintenance* enables researchers to ascertain key considerations for dissemination of the intervention beyond the trial – crucial for the success of programmes found to be effective in controlled settings. The use of RE-AIM to structure process evaluations facilitates understanding of both internal and external validity of an intervention combining assessment of uptake and effectiveness in the potential target population with potential for whole system-level delivery.

The Action 3:30R intervention is underpinned by Self-determination theory (SDT); a framework in which the quality of motivation is a key determinant of cognitive, affective and behavioural outcomes [14]. Autonomous motivation (i.e. doing PA because it is fun, satisfying or personally valued) versus controlled motivation (i.e. being active to comply with someone else’s demands or to avoid guilt) is associated with positive PA outcomes in children and adolescents [15]. Children’s motivation is likely to be more autonomous when they feel that three psychological needs are met: 1) autonomy (i.e. having choice and ownership over one’s behaviour, 2) competence (i.e. feeling able and confident in one’s abilities) and 3) relatedness (i.e. feeling a sense of mutual connectedness and belonging with others). Therefore, developing programmes that train TAs to promote autonomous motivation, may positively impact children’s motivation for, and engagement in, physical activity [16]. As the Action 3:30R intervention involved a complex and nuanced blend of teacher-pupil rapport, physical environments and school culture, a process evaluation exploring TA and pupil experience and the environmental (social and physical) factors at play in each school is essential to understand how and why the intervention was or was not effective.

### The aims of this process evaluation were

1. To explore whether TAs were effective at promoting autonomous motivation (theoretical fidelity).
2. To test the usefulness of the RE-AIM framework in the evaluation of a complex intervention.
3. To identify factors that may have affected the outcome results of the feasibility trial.
4. To explore the potential impact of the Action 3:30R intervention beyond the primary trial outcomes.

## Methods

The full trial methods [17] and feasibility trial results are presented elsewhere [18]. Briefly, 335 (*n* = 170 intervention, *n* = 165 control) pupils aged 8–10 years (mean age 8.4 ± 0.66, 49% female) were recruited from 12 primary schools across Southwest England. Six schools, stratified by local authority and percentage of pupils eligible for free school meals (an indicator of socioeconomic position), were randomly assigned to receive the refined Action 3:30R intervention, and six acted as non-intervention controls. In intervention schools TAs were trained to deliver a 15-week after school physical activity programme. Two schools did not deliver the intervention, meaning four intervention (*n* = 113 pupils) and six control schools (*n* = 139 pupils) were analysed at follow up. The feasibility study results showed that the intervention appealed to girls and lower active children and was inexpensive to deliver, so may be viable as an option for after-school physical activity provision in primary schools [18]. However, there was no evidence that it held promise to affect physical activity levels, with no difference in adjusted weekday MVPA between study arms at the end of the intervention (− 0.5, 95% CI = − 4.57, 3.57).

The process evaluation comprised both quantitative and qualitative methods. Quantitative measures were collected before and throughout the 15-week intervention to assess dose, fidelity and effectiveness of the intervention. Participation in other after-school clubs was also reported by parents. Qualitative measures, which consisted of focus groups and interviews, were conducted at the end of the intervention and aimed to collect views from a range of stakeholders. The process evaluation was structured according to the RE-AIM framework [13](Additional file 1). The measures are described in more detail in the sections below. Ethical approval was granted by The University of Bristol (ref: SPSREC16–17.B2). Written informed consent was obtained by all adult participants. Parental written informed consent was obtained for children [19] however children could make their own choice about taking part. Any child able to participate in PE lessons with informed parental consent was eligible to take part.

### The action 3:30R intervention

The Action 3:30 intervention has been revised since the previous Action 3:30 feasibility study [6], therefore this feasibility study and intervention is referred to as Action 3:30R. Two TAs from each of the intervention schools were required to attend 5 days (25 h) of training. TA training was designed in collaboration with, and delivered by, the Coach Development Manager at Bristol City Council who is a very experienced senior coach who has led training courses for a number of different sports. The training was designed to equip TAs to deliver Action 3:30R after-school clubs according to the Action 3:30R core philosophy which is underpinned by Self-determination theory (SDT); a framework in which the quality of motivation is a key determinant of cognitive, affective and behavioural outcomes [14]. The Action 3:30R after-school clubs were scheduled to run twice per week for 15 weeks. A re-enrolment point was offered to schools approximately half-way through the intervention, whereby new pupils were recruited to the study if spaces were available in the clubs due to pupils dropping out. Sessions were designed to be 60 min long and were intended to be delivered in the order they were numbered within the Leader’s Manual (containing the 30 pre-planned sessions) that each TA received. Sessions were designed to maximise child participation, skill development, cooperation, problem solving, physical activity and choice. TAs were trained to use an autonomy-supportive delivery style promoting the satisfaction of autonomy, relatedness and competence.

### Mapping to RE-AIM

The process evaluation used qualitative and quantitative approaches to map to RE-AIM components [20, 21] (Additional file 1). This was done to enable the reporting of each of the five RE-AIM elements and provided a simple structure that facilitated the interpretation of project findings. The definitions provided by RE-AIM [13] have been adapted and applied within the context of the Action 3:30R feasibility study, as shown in Table 1.

**Table 1:** Action 3:30R feasibility trial-specific RE-AIM definitions

RE-AIM component	Action 3:30R specific definition
Reach	The number, proportion and representativeness of individuals (TAs and pupils) who are willing to participate in Action 3:30R when offered the opportunity, as well as factors that may affect this
Effectiveness	The impact of Action 3:30R on the children, club leaders and schools involved, as well as factors which affected, or may affect this
Adoption	The number, proportion and representativeness of schools (settings) or TAs (agents) who are willing to initiate Action 3:30R, including factors that may affect this
Implementation	Absolute fidelity as well as factors affecting the fidelity of the delivery of Action 3:30R when compared with intended delivery, both at the individual level (TA adherence to protocols and session manual) and the structural level (school factors, resources, environment)
Maintenance	Factors perceived to affect potential maintenance of Action 3:30R

#### Reach

Reach was addressed in the process evaluation using quantitative data from the feasibility main trial on school and pupil recruitment rates, as well as registers of pupil attendance provided by each of the schools delivering the intervention. Average attendance by school and mean number and percentage of sessions attended by pupils were calculated. Semi-structured interviews conducted with TAs who delivered the intervention (*n* = 9) and key contacts who acted as the primary liaison between the school and study team (*n* = 7) as well as separate focus groups with boys (*n* = 6) and girls (*n* = 6) in each intervention school also provided qualitative evidence of factors affecting reach.

#### Effectiveness

Quantitative process measures were collected to address effectiveness from TAs before and after the five-day training and at three points during the 15-week intervention (November, January and March). TAs reported their efficacy to deliver PA sessions using an adapted version of the Physical Education Teacher’s Physical Activity Self-Efficacy Scale (PET-PASS) [22]. They rated the extent to which their delivery was autonomy-supportive using an adapted version of the Sport Climate questionnaire [23] and eight questions referring to a hypothetical teaching scenario [24]. At the same three time points, pupils reported their perceived exertion (11-point Likert scale: 0 (“not tired at all”) to 10 (“very, very tired”) and enjoyment (5-point Likert scale:1 (“not at all”) to 5 (“a lot”) of that session and a 6-item assessment of the TAs’ autonomy-support (7-point Likert scale: 1 (“strongly disagree”) to 7 (“strongly agree”) [23]. The focus groups with pupils and semi-structured interviews with TAs and school key contacts substantiated the quantitative data exploring training efficacy and the effectiveness of the intervention at fostering enjoyment of physical activity.

#### Adoption

Views on factors affecting adoption were elicited from TAs, school key contacts and pupils who took part in the intervention via the qualitative measures outlined above. Additionally, semi-structured interviews were conducted with external stakeholders who had expertise in primary school physical activity provision (*n* = 8) to explore potential factors affecting adoption from a public sector perspective.

#### Implementation

To assess adherence to key Action 3:30R principles and the specially-designed intervention session plans (fidelity), TAs completed a logbook after each session detailing whether they felt the pre-assigned session plan had been delivered fully, partially or not at all. At the three process measurement time points, two trained researchers also observed the sessions and took notes. Sessions were scored as being delivered ‘fully’, ‘partly’, or ‘not at all’, and reasons why were recorded. Questionnaire data are presented as overall mean and SD/SE at each interim time point. Attendance registers were used to assess intervention dose. Views on implementation fidelity, and factors affecting delivery such as training, resources and support, were elicited via the semi-structured interviews with TAs, school key contacts and the lead trainer who delivered the TA training, and the focus groups with pupils in each of the intervention schools.

#### Maintenance

Quantitative evidence of maintenance potential included any continued delivery beyond the end point of the intervention (30 pre-planned sessions). Qualitatively, views on factors affecting the decision to continue to deliver Action 3:30R and potential improvements to the intervention were obtained through the semi-structured interviews and focus groups with key participants as described in more detail below. Additionally, public sector perspectives on factors affecting the potential for maintenance were elicited through interviews with eight external stakeholders.

### Qualitative methodology and data analysis

Semi-structured interviews were conducted with TAs who delivered the intervention (*n* = 9), key contacts who acted as the primary liaison between the school and study team (*n* = 7), external stakeholders who had expertise in primary school physical activity provision (*n* = 8) and the person who trained the TAs (Lead Trainer) (*n* = 1). A paired-depth interview technique [25] was used for TAs. TAs in each school were interviewed together, to provide complete descriptions of events and opportunity to recall different experiences [26, 27]. Face-to-face interviews were conducted with all TAs, key contacts and class teachers and three of the eight external stakeholders. Telephone interviews were conducted with the other five external stakeholders. Separate focus groups, each lasting approximately 40 min, were conducted in school with boys (*n* = 6) and girls (n = 6) in each intervention school who were purposively selected to capture views from pupils with a range of attendance rates. Girls were asked specifically about participating with or without boys, and about how the intervention could appeal more to girls. Two researchers with Masters-level qualitative experience (one male, one female) conducted all the interviews and focus groups. Focus groups with boys were led by the male researcher and focus groups with girls were led by the female researcher to foster trust when discussing gender-sensitive topics. All informants received information about the research aims and researchers and provided written consent prior to interview. Field notes were made during and after each interview or focus group by the researcher present. The interviewer(s) pursued emerging themes and added these to the topic guides for subsequent interviews in an iterative process. Additional file 2 presents the recruitment details and topics covered for each participant group.

Interviews and focus groups were recorded using an encrypted voice recorder and transcribed verbatim. Two transcripts per participant group were initially coded independently by two researchers, who then met to discuss initial coding. Coding frameworks were then constructed for each participant group by four researchers (BT, AP, RJ, SS). The Framework Method [28] was used to analyse the data. Emerging inductive themes were explored as well as deductive themes, which were guided by the topic guides and underpinning intervention theory (SDT [14]). The pre-defined codes used in the deductive analysis were broad to allow for more refined codes and interpretations to emerge. Interviews and focus groups were imported into and analysed in NVivo (Version 11, QSR International Pty Ltd). BT and AP then applied the coding frameworks to the remaining transcripts and discussed any new codes that emerged. The coding frameworks were triangulated to compare codes between schools and participant groups and assess the degree of convergence. The qualitative methods are reported according to COREQ guidelines to facilitate credibility and transparency [29]. Quotes were anonymised and are presented with an identification (ID) (e.g. School 10 boy; School 7, TA; ES 2).

## Results

### Reach

#### Recruitment

Forty-four percent of schools initially contacted (12 out of 27) agreed to take part in the study [18]. Interviews with key contacts revealed that the main incentives for taking part were to add to current after-school provision and engage more lower active children, which aligned well with current school policies and/or priorities. Another reason voiced by one key contact was to offer parents a free after-school club.

**School 7, KC:** *because it’s a big thing at the moment to get children who are inactive active, we decided that we would want to take it on.*

Across all 12 schools in the study, 459 pupils returned parental consent to take part in Action 3:30R (41.39% of eligible), 49% of whom were girls. Across the four schools that delivered the intervention 111 pupils initially enrolled in the after-school clubs. Pupils expressed wanting to enrol in Action 3:30R to have fun, to be more active and to be with or make new friends. Pupils, TAs and key contacts suggested that commitment to other clubs and lack of parental engagement were the main barriers to recruitment.

**School 10, boy:** *Some people didn’t come, I think, because they had other after school clubs*.

**School 7, KC:** *You have to be face-to-face with some of the parents to try and persuade them to come on board ‘cause for whatever reason they are the parents that don’t engage so much and that’s why the children don’t come.*

#### Attendance

On average pupils attended 19 out of the 30 sessions and 73.5% of pupils attended more than half of sessions, with no difference in attendance between boys and girls. These figures are based only on pupils who attended at least one session and include re-enrolment pupils (*n* = 18), whose attendance rates were calculated by computing the proportion of sessions they could have attended from the point they were enrolled versus those they did. Table 2 presents the attendance figures by school.

**Table 2:** Mean number of sessions attended

School	n	Mean sessions attended	Mean sessions attended by boys	Mean sessions attended by girls	N pupils attending ≥50% of sessions	%
All^a, c^						
5	20	17.3	17.7	16.9	15	75.0
7	32	15.9	14.1	17.3	17	53.1
10	37	18.0	16.5	18.8	27	72.9
11	39	17.2	17.1	17.3	25	64.1
Total	128				84	
Average		17.1	16.3	17.3		66.3
Pupils who participated in at least one session^b, c^						
5	17	20.4	22.1	18.8	15	88.2
7	28	18.2	18.0	18.3	17	60.7
10	31	21.3	23.9	20.5	24	77.4
11	37	17.6	18.1	17.3	25	67.6
Total	114				81	
Average		19.2	20	18.7		73.5

n = number of pupils in the club

^a^‘All’ refers to all pupils enrolled into the study, including re-enrolment pupils and those who never attended a session

^b^Refers to pupils who attended at least one club session, including re-enrolment pupils and excluding pupils who did not attend any sessions

^c^Those who joined the club at re-enrolment are measured against the possible number of sessions they could have attended from the point re-enrolment was offered rather than the full 30

The average attendance numbers were similar before and after re-enrolment in all schools, suggesting that the re-enrolment point was effective at maintaining attendance levels. The qualitative findings suggest that key contacts and TAs felt attendance numbers were surprisingly high and consistent compared to their expectations, and, similar to recruitment, children were motivated to attend because the club was something fun to do with friends.

**School 10, TA:** *We didn’t have any issues with non-attendance… I would say our attendance was - blew us away, really.*

**School 11, girl:** *And it was like the people because everybody there was all kind and all the games were like really, really, really fun.*

Furthermore, a key contact highlighted that several regular attendees at their club were children who do not normally take part in after-school clubs, suggesting that the club appealed to a range of children and not just already-active pupils.

**School 7, KC:** *Really happy that they’ve got children who wouldn’t otherwise have gone involved but also that they, they’re staying ‘cause you don’t always see that either.*

After-school club scheduling conflicts was cited by pupils and TAs as the main barrier to attendance. When pupils were asked why they couldn’t attend all Action 3:30 sessions, many stated that they did other after-school activities, either in or out of school and because of this some children only attended one session per week.

**School 11, girl:** *I could only meet on the Wednesday because on Mondays I have swimming.*

**School 5, TA:** *You’ve got those that used to come on Tuesday but wouldn’t necessarily come on Thursday because youth club was running.*

A lack of parental support due to time restraints as well as the travel distance from home were suggested as other key barriers to attendance.

**School 10, KC:** *we get children from all over the place, so if they [parents] lived far away the thought of hanging around for an hour or coming back in an hour was a bit too much*.

### Effectiveness

The intervention effectiveness results have been reported elsewhere [18]. Briefly however, there was no evidence that when averaged across the week children in the intervention group did more physical activity, but they were more active on club days versus non-club days (mean 18.99 min of MVPA vs 10.38 min of MVPA respectively). In this section we draw on qualitative findings to explore whether the intervention worked to affect key outcomes, and what factors may have contributed to the results [20]. Specifically, we focus on the effectiveness of the intervention at promoting pupil enjoyment and TA efficacy to deliver autonomy-supportive PA sessions that promote self-determined motivation [15].

#### TA efficacy for delivering autonomy-supportive sessions

The five-day training course attended by TAs was received very positively. TAs liked the teaching style and delivery and valued and understood the theoretical underpinning, which focussed on creating a need-supportive learning environment [14].

**School 5, TA:** *Well, how to motivate children; not only in sport, in general. And it’s something that we can apply with any other session that we’re running with children; giving them that sense of belonging, giving them that sense of autonomy and all those things. That’s what I found interesting.*

**Fig. 1:**
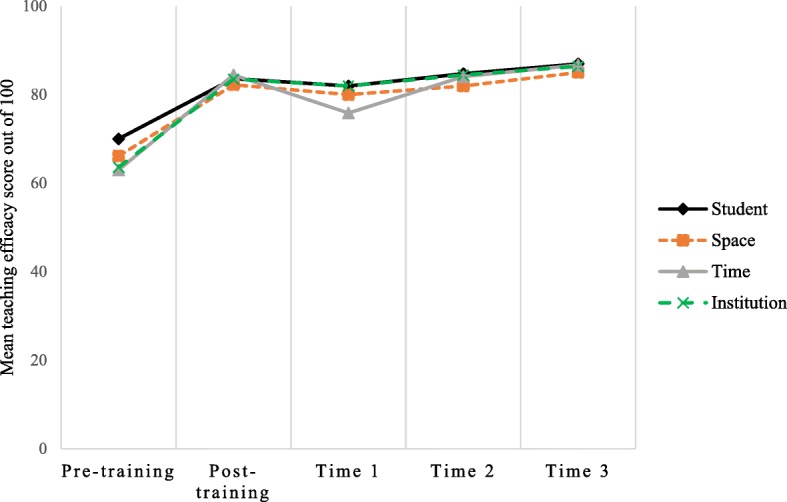
Self-perceived TA teaching efficacy at each process evaluation time point. Time 1, 2 & 3 refer to the 3 interim process evaluation observation visits conducted by research staff during the intervention period

The quantitative and qualitative results together suggest that TAs adopted an autonomy supportive-teaching style as a result of the training. Figure 1 shows that TAs’ confidence in dealing with issues relating to pupils, space, time and school support (institution) [22] all similarly increased from pre to post-training (mean = 65.67 to 83.44 out of 100) and then remained high throughout the intervention, with only a small decrease in time-related self-efficacy at process evaluation time point 1.

**Fig. 2:**
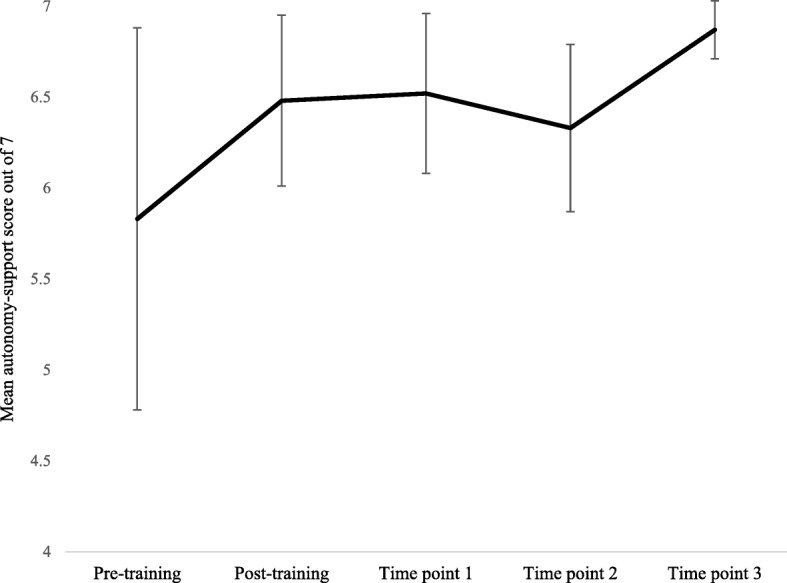
TA-perceived autonomy supportive teaching style at each process evaluation time point. Scores obtained using an adapted version of the Sport Climate questionnaire. Scale 1–7. Pre-training score, 5.83(1.05); Post-training score, 6.48(0.47); Time point 1 score, 6.52(0.44); Time point 2 score, 6.33(0.46); Time point 3 score, 6.87(0.16). Statistics presented are mean(SE) and error bars indicate the standard error

Figure 2 shows that the training increased TAs perceptions of using an autonomy-supportive style, which then remained relatively stable and increased further by process evaluation time point 3. Quantitative and qualitative pupil data corroborates the TAs delivery as autonomy-supportive. On average, pupils rated TAs’ teaching styles to be highly autonomy-supportive at all three process evaluation time points using the adapted Sport Climate questionnaire [23] (Means = 6.04, 5.91 and 5.97 respectively; scores are out of a maximum of 7). During focus groups, pupils characterised TA delivery style to be clear, fair, supportive and encouraging, balancing autonomy support with structure.

**School 5, girl:** *They were kind and supportive, but they were strict at the right times.*

Pupils also alluded to autonomy, competence and relatedness, the three needs underpinning self-determined motivation [14], by saying that they were offered plenty of choice about which games to play and how to adapt games to improve them, that they had become more confident in their physical and social skills, felt part of a team and felt cared for by the TAs.

**School 10, boy:** *They encouraged us to ask questions and tried to understand the way we wanted to do it*.

**School 5, girl:** *Because of the amount of fun we had and the connection between everyone and we would have great ideas and how our friendship would get better.*

In interviews, TAs also expressed that they felt that pupils had become more intrinsically motivated, as well as improved their physical and social skills.

**School 5, TA:** *It was extremely rewarding to see them change in so many different ways with their physical activity, with their skills, with the way that they became sports people and encouraging*.

#### Enjoyment

**Fig. 3:**
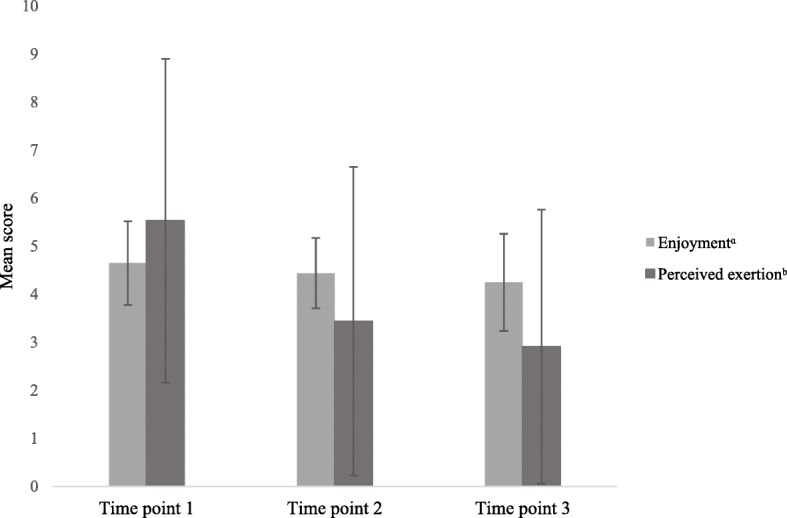
Mean pupil enjoyment and perceived exertion scores ^a^ score from 1 to 5. ^b^ score from 1 to 10. Mean(SD) enjoyment scores: Time 1, 4.63(0.87); Time 2, 4.44(0.73); Time 3, 4.25(1.01). Mean(SD) exertion scores: Time 1, 5.53(3.37); Time 2, 3.44(3.21); Time 3, 2.91(2.82)

Both the quantitative and qualitative results suggest that pupils enjoyed Action 3:30R throughout. Figure 3 shows that child-reported enjoyment was high at all three interim time points during the intervention. However, exertion was perceived to be relatively low.

Pupils stated that they enjoyed Action 3:30R because it was fun, included a variety of active games and child-led activities, was a mix of 2 year groups and because they were offered choice, which TAs agreed with.

**School 5, boy:** *It was good to see other’s ideas and it’s good to use them, and you get to like experience more things… like when people made up their own ideas, you’re basically like adding onto them to make their game better*.

**School 5, TA:** …*the one session that they actually really enjoyed is when they made their own games up. And then they’d say, ‘When are we going to do that?’*

Pupils mentioned very few things that they did not enjoy about Action 3:30R. The main barriers to enjoyment seemed to be disruptive behaviour (which would detract from time spent on activities) and lack of space to play big, active team games. There were mixed opinions among TAs about whether pupils enjoyed the skill-based activities; some pupils felt that pupils enjoyed seeing their own performances improve but others felt that pupils lost interest if activities were too challenging or had too many instructions.

**School 11, TA:** …*when it was completely new, something they had not done and there was quite a lot of rules…and ‘this is how you play it’… they lost interest quite quickly before we’d even got to the point of the starting the game*.

Girls seemed happy that the Action 3:30R club was a mixture of boys and girls, although some stated that boys could be disruptive at times. TAs felt that girls were as involved in the activities and able to express their opinions as the boys, which suggests that the intervention was effective in engaging and encouraging girls to be physically active.

**School 10, TA:** *I think we had enough strong-ish personalities in our girls that they’re not afraid to put their ideas out there and come forward.*

### Adoption

More than half of schools recruited to the study were above the local authority median for free school meals (indicator of familial socioeconomic position) suggesting that they represented catchments with a diverse range of socioeconomic backgrounds and were therefore representative of their region. In the four schools that delivered the intervention, adoption by TAs was excellent, suggesting that it is feasible for TAs to deliver an after-school physical activity programme. TAs stated that they either volunteered or were selected to be the delivery agents of the intervention. Interviews with key contacts in two of the control schools also revealed that they would have had no issues finding TAs willing to deliver the intervention. Key contacts and TAs considered the training to be a form of their professional training/development and so were willing to be out of school for 5 days.

**School 7, KC:** *as soon as they were approached, they were really keen to do it and they wanted to… We didn’t have to search around for staff at all.*

**School 10, TA:** *That’s the kind of thing that they sold it on, that you’d be going to a training course to run a club… As an experiment and that there’d be a qualification at the end of it.*

#### Barriers to adoption

Two schools allocated to the intervention arm were unable to deliver the intervention. An interview with the key contact in one of these schools revealed this was due to difficulty releasing staff to attend the training. The other school provided no information on why they withdrew from the study and no other data were provided. Therefore, more work may be needed at the school recruitment stage to understand school priorities and staff capacities.

Several external stakeholders perceived funding would be a main barrier to the adoption of Action 3:30R in schools that did not take part in the study because of the costs incurred to release TAs for training and to pay for delivery of the club. This was also echoed by a key contact in one intervention school.

**School 10, KC:** *I think the only way that would happen would be if we started charging the children, because the school doesn’t have any funds to fund teaching assistants to do clubs.*

The cost incurred to buy new equipment would also need to be considered. TAs expressed that the £200 given to schools to buy equipment was crucial for full delivery of the club. Sessions could be adapted to use existing equipment, however lack of funds to buy enough equipment could be a barrier in some schools.

Another barrier to adoption may be schools’ lack of capacity for additional after-school programmes. This was cited as a reason for not signing up by some of the schools initially contacted. Some ESs were also of the opinion that schools may already have enough after-school programmes.

**ES 2:** *I think at the moment primary schools are at a place where they’re building up their after-school offer on the whole. Some of them have felt they’ve probably reached capacity in some respects.*

#### Needs-related

External stakeholders purported that school targets, which are closely linked to current school needs would affect adoption. For example, some external stakeholders suggested that it is important to consider how a programme could generate whole-school outcomes in physical activity and well-being. This was also alluded to by key contacts and linked with the reasons schools signed up (Reach).

**ES 1:** *If we start with the programme itself and its relevance the key thing about sustainability is that it always has to deliver what the school needs… the key to sustainability for most schools is that it has to deliver whole school outcomes*.

#### Evidence related

The value of evidence to promote adoption was mentioned in many external stakeholder interviews, in a range of contexts. There is, however, divergence between different stakeholders about what evidence looks like. From a regional public health perspective, credibility gained through evidence of impact and affiliation to a respected institution or company were important factors to consider when deciding to commission a programme. However, from the school perspective, evidence of reliability and consistency may be more important to consider.

**ES 8:** …*we’d maybe look for that reliability and consistency. Because we have had companies that sent in different members of staff, you know with the children that’s not ideal.*

In line with this, as schools may already have good relationships with existing external coaching companies, in which they find reliable and trustworthy, several ESs suggested working with these companies, rather than offering schools programmes that replaces them, may lead to better adoption.

**ES 6:** *I think a conversation with a commercial provider, and I mean I don’t know if you’re open to a partnership approach almost… the coaching companies are in every school… they’ve already got some credibility so by buying in your product they would enhance what they’re delivering and it would also stop you having to think so much about how you can expand and grow.*

#### Practical considerations

Although TAs and key contacts in the four intervention schools stated that releasing staff for training was viable and that the format was appropriate, External stakeholders expressed concern that the time out of school would cause budgetary concerns and pressure on staffing structure, due to funding for TAs being cut in many local authorities.

**ES 2:** *Which is intensive [the training], which is great and I’m sure they got a lot from that but then that’s quite a commitment…*

Funding was a recurring theme throughout all external stakeholder and key contact interviews, which is also described in the *Maintenance* section. The external stakeholders believed that the PE and Sport Premium funding could be used to adopt and implement programmes like Action 3:30R, however not all key contacts mentioned this as an option, potentially due to the lack of guidance on how to spend the funding.

### Implementation

#### Dose adherence

All four intervention schools delivered all 30 sessions. The prescribed frequency and duration of the intervention was acceptable according to the TAs.

#### Content adherence

To capture adherence TAs were asked to complete a logbook of delivery. Of the planned sessions, TAs reported that 25% (*n* = 30) were delivered fully, 69% were delivered partially (*n* = 83), and 6% (*n* = 7) of session plans were not delivered at all. The reason for non-delivery of sessions was due to a temporary lack of space and so TAs chose to deliver a different session better suited to the available space. The evaluation of intervention fidelity was strengthened by observations conducted by trained researchers. Observed sessions were fully (*n* = 8, 67%) or partially (*n* = 4, 33%) delivered. Lack of time to complete the planned session components was observed as the reason for partial delivery.

### Factors affecting implementation fidelity

#### Training

The TA training was implemented as intended. According to the lead trainers’ logbook and observations by two researchers, all five training days were delivered as planned. Eight of the TAs attended all 5 days and one TA attended four. TA engagement during the training was excellent according to the lead trainer. In a post-training interview, he stated that they were one of the most engaged groups he had taught. He attributed this to well-explained course aims and a clear translation of Action 3:30R into relevant practice for the TAs – further evidence of training fidelity. In interviews, the lead trainer and TAs all agreed that there were no barriers to implementation of the training.

#### Resources

In addition to the Leaders’ manual, TAs were supplied with a Training Guide containing all the content from the training programme along with quick reference sheets signposting them to sections in the guide. Intervention schools were also provided with £200 to buy any equipment they needed to deliver Action 3:30R sessions.

The Leaders’ manual was highly valued by TAs because they did not have to pre-plan sessions. The only criticisms given by TAs were that there was often too much content to cover in each session and that some activities were difficult to visualise which could be improved by making some activities optional and adding explanatory diagrams.

**School 5, TA:** *It was very good because… We’d get it out and that’s what we would plan the session around and use throughout the session*.

**School 10, TA:** *We said about maybe a little diagram at the side would be helpful.*

#### School support

School-level support appeared to vary greatly between intervention schools. TAs in one school felt that very little school-level interest and support was given to their Action 3:30R club, which sometimes disrupted delivery.

**School 5, TA:** …*one session we had to go from the Key Stage 2 hall to Key Stage 1 because they organised with some life team or something to be in school and they never put any thought into that actually we were going to be using the hall.*

TAs in another school felt that sometimes there was a lack of communication with the administration staff with regards to club scheduling or attendance. The key contact at this school expressed that the autonomy demonstrated by the TAs running the club was valued.

**School 7, KC:** *They sort of just run with it. They went on their training. I checked in with them. They haven’t ever come to me and asked me anything, which has been fab because I’m really busy with my PE as well.*

Conversely, TAs in two schools felt well supported by other school staff, who aided delivery and promoted attendance when required.

#### Space and season

Space and season were cited most frequently by TAs as factors which affected the quality of intervention delivery. TAs felt that space was not big enough for some games, which pupils also agreed with.

**School 11, TA:** …*things like racket games, didn’t really work very well with the full group because there just wasn’t space.*

As Action 3:30R was delivered over the winter months, most sessions were conducted indoors, which meant space was more limited, sometimes the space had to be shared with other clubs when outdoor facilities were not in use and when sessions were conducted outdoors, they were sometimes limited for safety reasons.

**School 7, TA:** *There have been times, you know, when it was really torrential rain and I’ve said oh we might need the hall and they were just like, oh well tennis need the hall too, and we’d just have to make do with what it is*.

Although TAs stated that they were able to adapt the activities to suit their space, both TAs and pupils suggested delivering Action 3:30R in summer months (to use outdoor space more) as a potential improvement. Further evidence to support this is presented in the *Maintenance* section.

### Maintenance

Interviews with the key contact and TAs in School 7 revealed that they were continuing to run the Action 3:30R club twice a week for the remaining and following academic year. They also noted that they would make minimal changes to the programme. Key contacts and TAs in the other three schools also expressed an interest in delivering Action 3:30R again.

The intervention resources were believed to provide ample activities and ideas to use again and adapt, to enable the club to continue.

**School 10, TA:** *There’s enough games that fit together that you could just go on and on.*

TAs felt that the autonomy-supportive teaching style was paramount for the maintenance of the club. TAs expressed that they would continue to offer child-led activities. The key contact in School 7 planned to make this a focus of the club.

**School 7, KC:** …*it’s really great to be child-led I think…So I think that whatever works for the children… it’s probably good to try out those sessions first of all, see what the children like and then run with what they like. That’s probably how I’ll ask them to deliver it.*

#### Factors affecting maintenance

Although all four intervention schools expressed an interest in continuing Action 3:30R, several themes that may change the way the club is delivered or act as a barrier to future maintenance emerged.

#### Funding

Lack of funding and unsustainable funding were cited as the main barriers to maintaining Action 3:30R post-study by some school contacts. Identifying a funding source was challenging and some key contacts believed that it may be more financially viable if the club were to only run once per week. Concerns were raised about funding programmes such as Action 3:30R from time-limited sources (e.g., PE and Sport Premium).

**School 11, KC:** …*at the moment we have this Sport Premium funding from the government and so we’ve got quite a lot of money to be able to spend. It’s something that after 2020 we wouldn’t be able to fund quite easily.*

The key contact and TAs in another school considered charging parents to cover the delivery costs as the only viable option.

**School 10, KC:** *I don’t think it could be a twice a week thing because I don’t think you’d be able to charge enough to cover all of that… But then parents do pay for clubs and so it may well be something that they think about doing*.

Despite this perceived barrier, school 7 was able to deliver the club beyond the trial since they had decided to allocate some of their PE and Sport Premium funding to pay the TAs to continue it, so this barrier may be more or less relevant depending on school priorities.

#### Training

The training model used in Action 3:30R was valued by TAs and key contacts because it offered development opportunities to existing staff. External stakeholders also believed that training TAs increased the sustainability of the programme and therefore would appeal to other schools.

**ES 5:** …*the key point, is that the schools are encouraged to look at the sustainability model. And that CPD [continued professional development] is crucial to that*.

However, external stakeholders discussed how turnover of trained staff was a risk. It was suggested that a train-the-trainer model might increase the programmes’ sustainability.

**ES 7:** *So often just training two people is not sufficient, you need to maybe train four and keep them involved on a rotating basis in the programme if possible. So, you keep the skills up but you also keep a number of people involved and whether there’s some sort of train the trainer so those who are already there, if somebody new starts after the funding runs out, they have got a model to take it forward.*

#### Potential improvements

The three main themes that emerged from the data were *Space and season, Parental engagement* and *Creating peer leaders*. These themes are presented and described in Additional file 3.

## Discussion

This process evaluation set out to assess the feasibility, internal and external validity of the revised Action 3:30 intervention using the RE-AIM framework. The results show that it is feasible to recruit and train TAs to deliver an after-school physical activity programme using an autonomy-supportive teaching style. This may be appealing to primary schools because of the development opportunities offered, which aligns with current school targets of developing staff and expanding opportunities to cater to all students [30]. Table 3 summarises the main results under the RE-AIM headings.

**Table 3:** Summary table of main results under RE-AIM headings

Reach	44% of approached schools signed up. School contacts reported that a key motivation to sign up was to engage less-active pupils.
	The programme appealed to girls as much as boys; 41% of eligible pupils provided parental consent to participate in Action 3:30R, 49% of whom were girls.
	74% of pupils attended at least 50% of sessions. Scheduling conflicts with other clubs was the main barrier to attendance.
Effectiveness	The TA training was effective at increasing TAs confidence to deliver sessions and their adoption of an autonomy-supportive teaching style.
	Pupils and TAs found Action 3:30R sessions highly enjoyable. Pupils especially enjoyed the more autonomy-supportive delivery style and child-led elements.
Adoption	Four of six schools adopted the programme. The main perceived barriers to adoption in the other two were capacity of staff and, potentially, financial implications of providing cover for staff to attend TA training.
	Adoption is likely to be driven by whether the programme can generate whole-school outcomes in line with current priorities.
Implementation	TA training was delivered with high implementation fidelity.
	All four intervention schools delivered all 30 sessions.
	Session content was tailored by TAs in 75% of all sessions to adapt to their situations, demonstrating good adherence to the training principles.
	School support varied between intervention schools which may have impacted scheduling and attendance in two of the four intervention schools.
	Season of delivery affected how well some sessions could be delivered, due to space constraints of indoor spaces.
Maintenance	Unsustainable funding was cited by other schools as a primary barrier to continued delivery, however schools admitted that funding models such as charging parents were a possibility.
	One school reported continuing Action 3:30R beyond the trial despite withdrawal of study support and project funding, suggesting a funding barrier may be more/less important depending on school priorities.
	The TA training was a highly valued component that increased the potential sustainability of Action 3:30R and appealed to schools from a staff development perspective.

Globally, despite good intervention fidelity, recruitment and retention of pupils across the PA spectrum, results pertaining to the effectiveness of the intervention to increase weekday MVPA showed no difference overall between control and intervention groups at T1 [18]. Findings from this process evaluation suggest that one reason for this lack of effect could be that scheduling conflicts with other clubs meant that pupils were swapping one active club for another rather than adding activity where none existed before.

This study, alongside previous work [6], has shown that an after-school physical activity programme delivered by TAs can be feasibly implemented within primary schools. The five-day training model used to equip TAs with the knowledge, resources and qualification to deliver the Action 3:30R after-school club received very positive feedback from the TAs and key contacts in all four schools that delivered the intervention. The main incentive for attending training was continuous professional development (CPD), as the training would give confidence to TAs to continue to deliver extra-curricular physical activity beyond the study. TAs do not routinely receive training in physical activity provision and as such the Action 3:30 approach is novel and shows that TAs delivering physical activity programs is a viable method of delivery that is likely to be more cost-effective than using external sport coaches [18]. With the recent doubling of the PE and Sport Premium in England, primary schools are now expected to improve and expand their physical activity provision [30]. A recent Department of Education report suggests that schools should ensure that their provision is sustainable [31]. Schools have reported that providing existing staff with opportunities for CPD would help to achieve sustainability [32] and this is also consistent with Ofsted recommendations [33]. Our qualitative results revealed that one intervention school decided to continue to deliver Action 3:30R even after the project support had been removed, which suggests that Action 3:30R is sustainable beyond the completion of the study if schools elect to provide support for it. In addition, the key contact stated that the schools’ PE and Sport Premium would be used to fund the continuation.

Although there is evidence that the TA training may be appealing to schools [32, 34], our findings also showed that the training was a barrier to some schools adopting Action 3:30R. Two schools allocated to the intervention did not nominate TAs to attend the training and therefore were unable to deliver the intervention. Qualitative data revealed that difficulty in releasing staff for training was the main barrier in one school. External stakeholders also stressed that a five-day training model may be too time-intensive and financially unviable. The Department of Education report showed that from 2012/13 to 2014/15 (when PE and Sport Premium was first introduced), there was a large increase in the number of schools that employed external sports coaches to deliver extra-curricular PA programmes from 57 to 90%, whereas TA delivery only slightly increased (20 to 28%) [31]. Primary schools may therefore choose to pay for external sports coaches to deliver after-school PA programmes rather than increase workloads of existing staff, despite the potentially greater cost.

A key aspect of the training was its theoretical underpinning. Self-determination theory [14] was used to develop the training resources and teach TAs to adopt an autonomy-supportive teaching style. Using an autonomy-supportive teaching style is associated with increased feelings of autonomy, relatedness and competence in children, which in turn are associated with higher intrinsic motivation for and participation in PA [35]. The TA training was effective at increasing TAs’ teaching self-efficacy and perceived autonomy-support. These also remained relatively stable at each of the three process evaluation time points, suggesting TAs adopted an autonomy-supportive teaching style throughout the intervention and remained confident in their ability to deliver Action 3:30R under challenging circumstances. Compared to the previous Action 3:30 feasibility trial [6], TA teaching efficacy in all four domains (student, space, time and institution) were similar pre-training but higher at the end of the intervention, which may suggest that training had been effectively improved since the previous trial.

The results from this study show that we were able to recruit and retain boys and girls, which is important as low levels of PA among girls is an increasing concern. Specifically, we showed that 49% of pupils recruited to the study were girls and that all girls attended over half of the sessions. This evidence suggests that Action 3:30R was effective at appealing to and engaging girls in physical activity. Previous research suggests that enjoyment and perceived competence are important determinants of girls’ physical activity [36, 37]. Our process evaluation results showed that on average, enjoyment was rated highly by pupils attending Action 3:30R. Pupils were purposefully selected for the focus groups and pupils with varied attendance rates, including low attendance and re-enrolment pupils from each school, were selected to capture a range of views. The qualitative evidence suggested that girls enjoyed Action 3:30R and were equally as involved as the boys. Girls also alluded to increased competence as a result of taking part in Action 3:30R. Action 3:30R could be recommended to schools as a programme which is enjoyable and engaging for girls as well as boys.

Evidence of engagement was demonstrated by the high attendance rates. As a result of the previous Action 3:30 process evaluation [32], attempts were made to increase levels of attendance. Our quantitative results present evidence of relatively high attendance. Overall, on average 74% of pupils attended more than 50% of the sessions, with two schools achieving over 80%. These findings therefore provide evidence of good reach and fidelity and show that Action 3:30R is a programme ideally suited to use in school settings. Stakeholders including school contacts and commissioning bodies conveyed that an evidence base is an important consideration for the adoption of new programmes, however descriptions of evidence varied and the extent to which the evidence informs adoption was not clear from interview data. References were made to after-school scheduling conflicts and a market saturated with external providers, some of whom have pre-existing relationships with schools. These data therefore imply that efforts to capitalise on existing relationships with providers and to maximise the quality of abundant existing provision options may be a more valuable means of increasing physical activity than creating new options. As such, there is a need to identify the types of current provision, the quality of that provision and how it could be improved.

A strength of this paper is that we have reported an extensive and robust mixed-methods process evaluation using the RE-AIM framework [13]. RE-AIM helped us to understand whether Action 3.30 is acceptable in terms of uptake, the size of the target population (locally, nationally). It also addressed whether the school system can adopt, implement, afford and maintain Action 3.30, given the infrastructure, resources and variation in cost of delivery across schools [13, 21, 38] and provided a structure for conceptualizing and reporting research findings. Direct observations were also used to strengthen assessment of intervention fidelity. A number of participant groups were interviewed, including a range of well-informed external stakeholders, who were able to provide valuable insight into the sustainability of clubs like Action 3:30R. Pupils selected for the focus groups had varying attendance rates, therefore a range of views were likely captured. A major limitation of the study is that two schools were unable to deliver the intervention. One of these schools did not agree to an interview and therefore the reasons for dropping out of the study are unknown.

## Conclusions

In conclusion, our process evaluation, which has addressed all five components of the RE-AIM framework [13], suggests that Action 3:30R was implemented with high intervention and theoretical fidelity and demonstrates evidence of maintenance potential. The continuous professional development nature of the programme and relatively low cost were perceived to be key elements of Action 3:30R which suggest it would be a viable option for schools. Action 3:30R could be adopted by primary schools as an after-school physical activity programme which engages a range of pupils, including less-active children and girls, offers professional development for existing staff, and is enjoyable for pupils. More work is needed to make the training accessible for more primary schools. Researchers conducting process evaluations of school-based interventions should consider using the RE-AIM framework as it provided a helpful structure.

## Additional files

Additional file 1: How the process evaluation data collected maps onto components of the RE-AIM framework. (DOCX 14 kb)

Additional file 2: Descriptive information for each stakeholder group. (DOCX 14 kb)

Additional file 3: Potential improvements for the maintenance of Action 3:30. (DOCX 14 kb)

## Data Availability

The datasets and supporting materials for the current study are available in the University of Bristol Action 3:30R data archive; link available from the corresponding author on reasonable request.

## Abbreviations

CPD: Continuous professional development
ES: External stakeholder
KC: Key contact
MVPA: Moderate to vigorous physical activity
PA: Physical activity
SDT: Self-determination theory
TA: Teaching assistant
