# Supplementary material for: Action 3:30R: process evaluation of a cluster randomised feasibility study of a revised teaching assistant-led extracurricular physical activity intervention for 8 to 10 year olds
Source: BMC Public Health. 2019 Aug 14;19:1111. doi: 10.1186/s12889-019-7347-3 (PMC6694632; doi:10.1186/s12889-019-7347-3)
Supplement: Supplementary file 1 — How the process evaluation data collected maps onto components of the RE-AIM framework. (DOCX 14 kb) [file 12889_2019_7347_MOESM1_ESM.docx]

## Additional file 1. How the process evaluation data collected maps onto components of the RE-AIM framework

| **RE-AIM component** | **Data collected** | **Type of measure** | **Description** |
| --- | --- | --- | --- |
| **Reach** | School recruitment  Pupil recruitment  TA registers  Views on recruitment and attendance | Quantitative  Quantitative  Quantitative  Qualitative | % of schools willing to take part  % of pupils who consented to study  Measure of pupil attendance  Interviews with TAs and KCs, focus groups with pupils |
| **Effectiveness** | Child enjoyment  Child perceived exertion  Child perceived autonomy-support  TA perceived autonomy-supportive teaching style  Views on the enjoyment and theoretical underpinning of intervention | Quantitative  Quantitative  Quantitative  Quantitative  Qualitative | Questionnaire  Questionnaire  Questionnaire  Questionnaire  Interviews with TAs and KCs, focus groups with pupils |
| **Adoption** | Views on factors affecting adoption | Qualitative | Interviews with TAs, KCs and external stakeholders |
| **Implementation** | TA log books  Observational visits  TA training register  Views on intervention delivery, training, resources and factors affecting fidelity | Quantitative  Quantitative & Qualitative  Quantitative  Qualitative | % of sessions delivered fully, partially or not at all  Trained researchers observed sessions and assessed delivery  TA attendance during five-day Action 3:30 training course  Interviews with TAs, KCs and Lead Trainer, focus groups with pupils |
| **Maintenance** | Views on factors affecting the decision to continue to deliver Action 3:30 and potential improvements | Qualitative | Interviews with TAs, KCs and external stakeholders, focus groups with pupils |
