# Supplementary material for: Action 3:30R: process evaluation of a cluster randomised feasibility study of a revised teaching assistant-led extracurricular physical activity intervention for 8 to 10 year olds
Source: BMC Public Health. 2019 Aug 14;19:1111. doi: 10.1186/s12889-019-7347-3 (PMC6694632; doi:10.1186/s12889-019-7347-3)
Supplement: Supplementary file 2 — Descriptive information for each stakeholder group. (DOCX 14 kb) [file 12889_2019_7347_MOESM2_ESM.docx]

## Additional file 2. Descriptive information for each stakeholder group

| **Participant group** | **Recruited vs approached** | **Description of participantsª** | **Themes covered in topic guides** |
| --- | --- | --- | --- |
| Teaching assistants  (TA) | 9 out of 9 | 3 INT schools with 2 TAs  1 INT school with 3 TAs | Content and delivery of training, managing disruptive behaviour, attendance, delivery, enjoyment and theoretical fidelity of intervention and potential improvements |
| Key contacts  (KC) | 7 out of 7 | N=1 Business administrator (INT school)  N=1 P.E coordinator (INT school)  N=2 P.E coordinator/class teacher (1 INT/1 CON school)  N=2 Class teachers (INT schools)ᵇ  N=1 Deputy Head teacher (CON school) | Recruitment, delivery and enjoyment of intervention, potential to continue Action 3:30, logistics, burden and potential improvements |
| External stakeholders (ES) | 8 out of 12 | N=4 representatives from local authorities  N=1 primary school PE coordinator  N=3 representatives from national bodies | Sustainability, future commissioning potential, dissemination opportunities and potential improvements |
| Pupils | 48 out of 48 | **For each intervention school:**  N=2 girls in the highest third of attendance  N=2 boys in the highest third of attendance  N=1 girl in middle third of attendance  N=1 boy in middle third of attendance  N=2 girls in lowest third of attendance  N=2 boys in lowest third of attendance  N=1 girl who joined at re-enrolment  N=1 boy who joined at re-enrolment  1-2 reserve pupils per school selected in case of absence on the day | Recruitment, attendance, delivery and enjoyment of intervention and potential improvements |
| Lead Trainer | 1 out of 1 | Qualified coach who taught majority of the training content to TAs | Training fidelity, perceived TA enjoyment and ability, potential improvements to training |

ªINT=intervention school, CON=control school.

ᵇincludes one key contact from a school that was allocated to but not retained to the intervention group.
