# Supplementary material for: Action 3:30R: process evaluation of a cluster randomised feasibility study of a revised teaching assistant-led extracurricular physical activity intervention for 8 to 10 year olds
Source: BMC Public Health. 2019 Aug 14;19:1111. doi: 10.1186/s12889-019-7347-3 (PMC6694632; doi:10.1186/s12889-019-7347-3)
Supplement: Supplementary file 3 — Potential improvements for the maintenance of Action 3:30. (DOCX 14 kb) [file 12889_2019_7347_MOESM3_ESM.docx]

## Additional file 3. Potential improvements for the maintenance of Action 3:30

| **Potential improvement** | **Description** | **Supporting qualitative evidence** |
| --- | --- | --- |
| Space and Season | Deliver Action 3:30 in the Spring/Summer months so that outdoor facilities can be used, there is more room for big group games and less concerns for safety. | *I think it would also be really nice to do, instead of in the winter months, do it spring/summer months. I think that would be really nice. I wondered maybe even if more children would attend those months* (TA, School 25)  *Maybe if we did it again, I would like to go outside because there was more space and less people get hurt* (boy, School 25) |
| Parental engagement | Try to engage parents more, by involving them in recruitment and having face-to-face conversations. Potentially involving parents in the delivery of the club as volunteers, however this is likely to only engage the more proactive parents. Parental engagement was raised as a barrier to recruitment and attendance by TAs and KCs, however findings from this process evaluation do not highlight many ways to combat this. This should be a focus for future primary school-based interventions. | *You have to be face-to-face with some of the parents [yes] to try and persuade them to come on board ‘cause for whatever reason they are the parents that don’t engage so much and that’s why the children don’t come* (KC, School 27)  *You could have like a taster session or something the kids could come to and parents could maybe take part in as well* (TA, School 33)  *So they're both engaging parents as volunteers within the delivery, but they're also then taking the content home and sustaining that movement, that activity level beyond the school gates* (ES 1) |
| Creating peer leaders | One ES recommended involving children who have previously been involved in Action 3:30 to help deliver the club. This may act to promote child ownership and achieve sustainability. One KC also mentioned this as a strategy that the school may consider when continuing the club. | *The tipping point for us, or the landmark point for us, was when we gave ownership of the delivery of the clubs to children… Year 6 pupils who had ownership of the club, the delivery of the club, and were able to tinker with the activities and the way in which they were delivered and provided almost like a buddy relationship with the children that were involved and at that point the clubs really exploded, not just in terms of the amount that were delivered but, you know, even consistently now there's still over eight and a half thousand clubs running and they are largely due to the fact that they are delivered by children for children.* (ES 1)  *Maybe we might even make some leaders because we’ve done that before, so some of the children who are doing it now could then go and help* (KC, School 27) |
